# Leaching behaviour of pendimethalin causes toxicity towards different cultivars of *Brassica juncea* and *Brassica campestris* in sandy loam soil

**DOI:** 10.2478/v10102-009-0025-z

**Published:** 2009-12-28

**Authors:** Subhendu Bandyopadhyay, Partha P. Choudhury

**Affiliations:** 1 Department of Agronomy, Uttar Banga Krishi Viswabidhyalaya, Pundibari-736165, Coochbehar, West Bengal, India; 2 Department of Biochemistry, UBKV, Coochbehar, India

**Keywords:** rape seed, mustard, pendimethalin, dinitroaniline, phytotoxicity, leaching

## Abstract

An experiment was conducted at the farm of Zonal Adaptive Research Station, Uttar Banga Krishi Viswavidhyalaya, Pundibari, Cooch Behar, West Bengal to evaluate the effect of pendimethalin on the yield, weed density and phytotoxicity in different varieties of rai (*Brassica juncea*) and yellow sarson (*B. campestris* var. yellow sarson) under higher soil moisture regime in *Terai* region of West Bengal. Pre-emergence application of pendimethalin at higher dose i.e. 1.0 kg/ha recorded higher plant mortality (30.92%) due to the presence of higher concentration of pendimethalin residue (0.292 µg/g) till the tenth day of crop age and consequently had the reduced yield (12.59 q/ha) than the dose of 0.7 kg/ha (13.33 q/ha) where plant mortality was only 12.62% due to comparatively lower level of pendimethalin residue (0.192 µg/g). Although the application of pendimethalin at the rate of 1.0 kg/ha was able to control weed more efficiently (18.96/m^2^) than the dose of 0.7 kg/ha (30.41/m^2^) and subsequent lower doses. The herbicide leached down to the root zone resulting in phytotoxicity towards crop. Yellow sarson group (*Brassica campestris*) showed more susceptibility than rai (*Brassica juncea*) group against pendimethalin application at higher doses.

## Introduction

Rapeseed-mustard is one of the most important oilseed crop grown extensively in the northern part of West Bengal in India. In *rabi* season higher infestation of broadleaved weeds like, *Chenopodium album*, *Gnaphalium purpurium*, *Polygonum spp*. etc. and severe infestation of *Stelaria media*(common name: chick weed, family: Caryophyllaceae) accounting for higher cost of cultivation with manual weed control measure, forcing the resource scare farmers to switch over to the other crops. In that case, inclusion of herbicides in integrated weed management schedule is the pre-requisite to have the effective weed control as well as to reduce the cost of manual weeding. Application of pendimethalin [N-(1-ethylpropyl) 3, 4-dimethyl-2, 6-dinitroaniline], a pre-emergence herbicide may be the better proposition. Pendimethalin is a non-ionic dinitroaniline herbicide used for the selective control of grassy and broadleaf weeds in a variety of crops (Sinha et al., 1996; Tsiropoulos and Miliadis 1998; Bhowmick and Ghosh 2002). It can control all the weeds present in mustard field under North Bengal condition. But visual observation at farmers' field showed that application of pendimethalin at recommended dose of 1.0 kg/ha caused crop plant phytotoxicity in lighter sandy loam soil of *Terai* region of West Bengal particularly after receiving a substantial amount of rain following the application of herbicide. Pendimethalin is a low volatile and low mobile herbicide having low water solubility (Savage and Jordan 1980; Schleicher et al. 1995). It is moderately persistent with a field half life of approximately 30 days and lateral and downward movement is restricted (Lee et al., 2000). It does not go rapid microbial degradation. Slight loss can occur from photodecomposition and volatilization. It is strongly absorbed by moist soil, practically insoluble in water and thus does not leach appreciably in moist soil (Aktar et al., 2008). Signori and Deuber (1979) revealed the higher leaching of pendimethalin in loamy soil than in clay soils.

Keeping this in view the field experiment was conducted at the farm of Zonal Adaptive Research Station, Pundibari, Uttar Banga Krishi Viswabidyalaya, Cooch Behar, West Bengal to standardize the optimum dose of pendimethalin in *Terai* agroclimatic situation of West Bengal and to determine the extent of phytotoxicity in different varieties of rai/yellow sarson grown in the soil of this farm at various doses of pendimethalin.

## Materials and methods

### Chemicals

A formulation of pendimethalin 30% emulsifiable concentrate (Stomp 30 EC) was procured from the market. An analytical grade of pendimethalin (Purity 99.6%) was supplied by AccuStandard. Solvents, viz. hexane, acetone, chloroform of analytical grade and other chemicals were procured locally from E. Merck Company. All the solvents were distilled and dried before use.

### Field trials and design

The field experiment was carried out in the winter season of 2006–07 on a sandy loam soil (Table 1) at the farm of Zonal Adaptive Research Station, UBKV, Pundibari, Coochbehar, West Bengal, India (26°50′ N; 88°83′ E). To assess the level of phytotoxicity of pendimethalin on mustard, four varieties, viz. B-9, NC-1, Pusa Bold and Baruna of rai/yellow sarson were chosen. The experiment was laid out in a split-plot design with three replicates and individual plot of 12 m^2^. The main plots were assigned as four levels of different doses of pendimethalin (D_0_: 0.0 kg/ha; D_1_: 0.35 kg/ha; D_2_: 0.70 kg/ha and D_3_: 1.0 kg/ha.), where as in the sub-plots four varieties of rai/ yellow sarson were assigned [V_1_: B-9 (yellow sarson); V_2_: Nc-1 (yellow sarson); V_3_: Pusa Bold (Rai) and V_4_: Baruna (Rai)]. All the plots received 60 kg nitrogen (N) /ha, 40 kg phosphorus pentoxide (P_2_O_5_) /ha and 40 kg potassium oxide (K_2_O) /ha. Pendimethalin at assigned doses were applied after 1 day of sowing.

**Table 1:** Physiochemical properties of the soil collected for residue analysis.

Properties	Surface Soil
Depth from top (cm)	0–15
Texture	sandy loam
Particle size distribution	
fine sand (%)	69
silt (%)	21
clay (%)	10
pH (soil to water; 1:2.5)	6.0
Maximum water holding capacity (%)	45
Organic carbon (%)	0.65

### Pendimethalin residue analysis

#### Sampling

Soil samples (0–30 cm depth, 500 g) were drawn randomly using a 2.5 cm diameter tube auger from 6 spots in each plot on 0 (2 h), 1, 10, 45 days after treatment (DAT). Samples were mixed thoroughly, air-dried, ground and passed through 2 mm sieve. A representative 50 g sample was taken by quartering for analysis.

#### Extraction and cleanup

A single step extraction and clean-up method was adopted (Kulshrestha *et al*., 1971; Raj *et al*., 2003). Soil sample of 25 g mixed with 0.5 g each of activated charcoal and florisil was filled in a 30 cm long glass column having 1.8 cm i.d. The residues were eluted with 150 mL of n-hexane: acetone (1:1). The organic phase was evaporated to dryness in rotary vacuum evaporator. Final volume was made-up by n-hexane.

#### Quantification

The residues extracted in n-hexane were analysed by GLC using Hewlett Packard Model 5890A series II equipped with ^63^Ni ECD fitted with HP-17 megabore column. Nitrogen was used as carrier gas maintaining the flow rate of 25 mL/min. The oven, injector and detector temperatures were 250, 300 and 210 °C, respectively. Following this condition the retention time was found to be 1.3 min.

#### Recovery

To work out the extraction efficiency of methods employed for pendimethalin from soil, sample matrices were spiked in triplicate at two different levels (i.e. at 0.25 and 0.50 µg/mL) with the above mentioned herbicide. The average recoveries of pendimethalin were 85 and 92 percent.

### Soil moisture studies

To resemble the situation in the farmers' field after receiving the substantial amount of rain and to observe the efficacy of pendimethalin under high soil moisture condition, a pre sowing irrigation (5 cm) was given. The soil moisture percentage at 0 (2 hr), 1, 10 and 45 days after treatment of herbicide was calculated by gravimetric method as proposed by Dastane (1974).

### Phytotoxicity study

#### Weed counts

Total weed density was assessed using a 0.25 m^2^ quadrant that was randomly placed within the plots after 30 days of sowing.

#### Crop plant mortality

The count on plant population have been taken after 7 days of sowing of seeds and continued till 26 days with an interval of three days.

#### Crop yield

The pod was collected from the core area of 3m × 2 m of each plot, barring 1 m from each side.

## Results and discussions

### Control of weeds

Application of Pendimethalin at higher doses was able to control more number of weeds/m^2^. Reduction of weed population was 78.5% and 86.6% in D_2_: 0.070 kg/ha and D_3_: 1.0 kg/ha, respectively over control.

### Crop plant phytotoxicity

Irrespective of variety, application of pendimethalin in higher doses induced the crop plant phytotoxicity (12.62% and 30.92% in D_2_: 0.70 kg/ha and D_3_: 1.0 kg/ha, respectively). The extent of damage was considerably higher in case of *campestris* group than the *juncea* group. That ultimately reflected on the yield of crops.

### Crop yield

As the higher doses of Pendimethalin controlled the weed efficiently, the yield corresponding to those plots were higher (higher by 119.6% and 107.4% in D_2_: 0.70 kg/ha and D_3_: 1.0 kg/ha, respectively over control) as expected. But on the other hand, application of extra 0.3 kg/ha of pendimethalin over 0.70 kg/ha induced the crop phytotoxicity and decreased the yield by 5.5%. Irrespective of dose of pendimethalin application, *juncea* group of mustard produced the higher yield.

### Soil moisture (%)

Irrespective of treatments applied, soil moisture decreased considerably with the passage of time in all the plots (Table 3). In the 0 DAT (i.e. one day after application of pre-sowing irrigation) the soil moisture varied from 39–41%, where as in the light soil of *Terai* zone of West Bengal just after a day the same came down to 25–30% and after 10 DAT that was 19–22%.

### Dissipation of Pendimethalin

The rate of dissipation of pendimethalin was very rapid (40–44%) in all the doses caused by pre-sowing irrigation. But 42 to 48% of applied pendimethalin persisted up to 10 DAT (Table 3). These available residues of pendimethalin had pronounced effects on weed control, phytotoxicity and yield of the crop.

### Regression analysis between soil moisture (%) and pesticide residue

**Figure 1:**
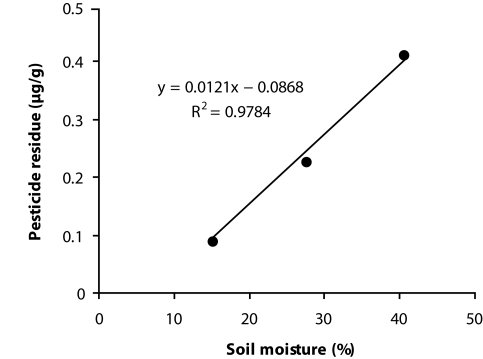
Regression analysis between soil moisture (%) and pesticide residue.

Regression analysis (Figure 1) showed the linear negative relation between soil moisture and soil pesticide residue. The higher R^2^ value (0.9784) established the significant responsibility soil moisture towards dissipation of pendimethalin. Bailey and White (1964) observed that most of the herbicides have higher phytotoxicity at higher soil moisture contents. They attributed this to the degree of competition of the organic compounds for the absorption sites at different moisture levels. Irrigation or rainfall following herbicide application has a profound effect on leaching and crop and weed tolerance to an herbicide. Rao (1983) expressed that in the light soil of *Terai* region combining with high moisture probably enhanced the leaching of pendimethalin which persisted till the 45 DAT at a detectable quantity (Table 2) to affect the comparatively deep rooted mustard crop.

**Table 2:** Effect of different doses of Pendimethalin on yield, weed density and phytotoxicity in different varieties of rai (*Brassica juncea*) and yellow sarson (*B. campestris* var. yellow sarson) in *Terai* agro-climatic condition.

Treatments	Yield (q/ha)	No. of weeds / m^2^ at 45 DAS	Crop plant mortality
**Levels of Pendimethalin**			
D_0_: 0.00 kg/ha	6.07	141.58 (2.151)^*^	2.232 (1.494)^**^
D_1_: 0.35 kg/ha	11.92	69.34 (1.341)	8.283 (2.878)
D_2_: 0.70 kg/ha	13.33	30.41 (1.483)	12.620 (3.552)
D_3_: 1.00 kkg/ha	12.59	18.96 (1.278)	30.920 (5.561)
S.E M. (±)	0.431	0.031	0.226
C.D. (0.05)	1.490	0.109	0.782
**Varieties of rai/ yellow sarson**			
V_1_: B-9	9.63	54.20 (1.734)	21.01 (4.584)
V_2_: NC-1	9.39	51.05 (1.708)	19.09 (4.369)
V_3_: Pusa Bold	12.50	46.24 (1.665)	4.63 (2.152)
V_4_: Baruna	12.38	44.15 (1.645)	5.66 (2.380)
S.E. M. (±)	0.617	0.041	0.224
C.D. (0.05)	1.801	NS	0.653

^*^ Figures in parenthesis are log (x) transformed value

^**^ Figures in parenthesis are √(x) transformed value; NS=Not significant

**Table 3:** Soil moisture (%) at different days after application of pendimethalin in soil of *Terai* agro-climatic region.

	Average soil moisture (%)			
	
Treatments	0 DAT	1 DAT	10 DAT	45 DAT
D_1_: 0.35 kg/ha	39.3	30.1	22.4	16.3
D_2_: 0.70 kg/ha	41.5	25.4	19.0	14.7
D_3_: 1.00 kg/ha	41.0	27.3	19.5	14.6

DAT: Days after treatment of pendimethalin

**Table 4:** Dissipation of Pendimethalin in soil of *Terai* agro-climatic region.

	Average residues (μg/g) at different days after application			
	
Treatments	0 DAT*	1 DAT	10 DAT	45 DAT
D_1_: 0.35 kg/ha	0.143 (0)	0.085 (40.56)	0.064 (55.24)	BDL*
D_2_: 0.70 kg/ha	0.400 (0)	0.210 (47.5)	0.192 (52)	0.094 (76.5)
D_3_: 1.00 kg/ha	0.683 (0)	0.380 (44.36)	0.292 (57.24)	0.170 (75.11)

DAT: Days after treatment of pendimethalin

BDL: Below detectable limit; Figures in parentheses express the percentage of dissipation of applied pendimethalin

## Conclusion

Pendimethalin can move downward to root zone of crop in light textured soil in presence of sufficient moisture (Chopra *et al*., 2009). This portion of pendimethalin can exert phytotoxic effect on crop. This phenomenon has been revealed in the present study and had been observed in farmers' field too. So, the determination of dose of this herbicide and application of irrigation are two major points to be considered. After the introduction of pendimethalin in mustard in this region the usual practice of farmers is to apply it at the rate of 1 kg/ha. This dose had created huge loss in crop yield due to severe phytotoxicity. From this study, a dose at the rate of 0.7 kg/ha may be proposed for light soil. Interestingly, *Brassica juncea* (Pusa Bold and Varuna) showed significantly less phytotoxicity towards pendimethalin than *Brassica campestris* (NC-1 and B-9). This information can well be utilized in crop development programme.
